# Improved Accuracy of the Asymmetric Second-Order Vegetation Isoline Equation over the RED–NIR Reflectance Space

**DOI:** 10.3390/s17030450

**Published:** 2017-02-24

**Authors:** Munenori Miura, Kenta Obata, Kenta Taniguchi, Hiroki Yoshioka

**Affiliations:** 1 Department of Information Science and Technology, Aichi Prefectural University, 1522-3 Ibara, Nagakute, Aichi 480-1198, Japan; id151003@cis.aichi-pu.ac.jp (M.M.); id151002@cis.aichi-pu.ac.jp (K.T.); 2 National Institute of Advanced Industrial Science and Technology, The Institute of Geology and Geoinformation, Central 7, 1-1-1, Higashi, Tsukuba, Ibaraki 305-8567, Japan; kenta.obata@aist.go.jp

**Keywords:** inter-band relationship, vegetation isoline, cross calibration, asymmetric, leaf area index (LAI), canopy RT model, inversion

## Abstract

The relationship between two reflectances of different bands is often encountered in cross calibration and parameter retrievals from remotely-sensed data. The asymmetric-order vegetation isoline is one such relationship, derived previously, where truncation error was reduced from the first-order approximated isoline by including a second-order term. This study introduces a technique for optimizing the magnitude of the second-order term and further improving the isoline equation’s accuracy while maintaining the simplicity of the derived formulation. A single constant factor was introduced into the formulation to adjust the second-order term. This factor was optimized by simulating canopy radiative transfer. Numerical experiments revealed that the errors in the optimized asymmetric isoline were reduced in magnitude to nearly 1/25 of the errors obtained from the first-order vegetation isoline equation, and to nearly one-fifth of the error obtained from the non-optimized asymmetric isoline equation. The errors in the optimized asymmetric isoline were compared with the magnitudes of the signal-to-noise ratio (SNR) estimates reported for four specific sensors aboard four Earth observation satellites. These results indicated that the error in the asymmetric isoline could be reduced to the level of the SNR by adjusting a single factor.

## 1. Introduction

Estimation of biophysical parameters from remotely sensed reflectance requires calibration [1], inter-comparison of reflectance spectra [2] and derived data products [3]. Parameter retrieval based on those calibration efforts has been a major goal of land analysis disciplines [4]. The outcomes of such efforts provide crucial information about local and global areal coverage, information that is used in a wide range of applications [5]. Although numerous investigations have reported the development and improvement of biophysical parameter retrieval algorithms, many of these algorithms involve simple algebraic band manipulations known as spectral vegetation indices (VIs) [6,7]. A variety of VI models have been investigated for their robustness against both internal and external influences [8,9,10,11,12,13,14,15].

A key component of VI model development is the relationship between two reflectances of different bands obtained under fixed biophysical parameter conditions. This relationship produces a reflectance spectrum trajectory in a reflectance subspace attributed to a fixed biophysical parameter value; therefore, this relationship is known as a vegetation isoline. The concept of a vegetation isoline has been used repeatedly to develop optimal VI models [8,9,12,16] and to investigate their robustness against external factors [17,18]. The isoline concept has been directly used to retrieve leaf area index and the fraction of vegetation cover [19,20]. In recent years, the concept has been applied to the inter-sensor calibration of VIs [21,22].

From the application point of view, understanding of band-to-band relationship would provide information about land cover dependency of calibration coefficients. In retrievals of biophysical parameter, an isoline equation with higher accuracy would lead to better results in retrieved parameters. Moreover, biophysical parameters vary along with the evolution of phenology, which eventually influence on reflectance spectra. Since the derived coefficients of the vegetation isoline depend on the biophysical parameters, the phenology is also related to the variation of the vegetation isoline.

Significant efforts have been devoted toward deriving useful analytical formulas based on a model of radiative energy transfer. These derivations used a representation of the top-of-canopy (TOC) reflectance spectrum consisting of photons that were directly reflected by the canopy layer. Because this portion of the reflectance spectrum does not reach the soil surface beneath the canopy, it is called the zero-th order interaction term. Photons that reached the soil surface and were reflected back to the canopy layer by the soil surface only once contributed to the measured reflectance. The ‘one-time reflected’ contributions comprised the first-order interaction term. Analogously, the reflectance spectrum consisting of photons reflected by the soil surface n times was defined as the n-th-order interaction term. The vegetation isoline equations were derived by truncating the second- and higher-order interaction terms. For this reason, the derived isolines are a first-order approximation of the vegetation isoline.

The approximation order determines the accuracy of the derived isoline equations. The accuracy of the isoline has been improved by deriving several approximations that considered the second-order terms. The accuracy has been improved by including higher-order terms. The drawback of this inclusion is that the analytical representation is complex. Complex representations hinder the employment of isoline formulations in applications of various types. It would be beneficial to identify ways of improving the isoline approximation accuracy while maintaining the simplicity of the derived formulation.

In a previous study, we proposed a derivation technique for satisfying these requirements simultaneously [23]. During the derivation, we included the second-order interaction term only in the near-infrared band instead of retaining the second-order term in the red band. This asymmetric approximation form dramatically improved the accuracy of the derived isoline equation.

This study advanced the investigation one step further. The objective was to introduce a technique for improving the accuracy of the asymmetric isoline equations by optimizing a single factor. The goal of this improvement was to reduce the errors in the vegetation isoline equivalent to a value equal to or smaller than the error induced by the inherent signal-to-noise ratio (SNR) of the existing sensors. The accuracy improvements obtained in this study were validated using a radiative transfer model of a system of vegetation and soil layers, the PROSAIL model [24,25]. After optimizing a single factor in the asymmetric approximation of the vegetation isoline equation, the error levels of the improved isoline equations are discussed by comparing the resulting errors with those computed directly from the signal-to-noise ratios of four existing sensors.

## 2. Background

In this section, two forms of the vegetation isoline equation are introduced. The magnitudes of the errors in the isoline equations differed between the two equations and were characterized numerically.

### 2.1. Two Approximations of the Vegetation Isoline Equations

The simplest form of the vegetation isoline equation was derived by truncating the soil–canopy interaction terms at the first-order (single interaction) [26]. The resulting equation was simple, which is advantageous for various applications [17,18,19,21,22,27,28,29,30,31]. The first-order isoline equation may be written (with the truncation term $\epsilon_{1}$) as:

(1) $$\begin{array}{c} \rho_{N}=a\gamma_{1}\rho_{R}+D_{1}+\epsilon_{1}, \end{array}$$

where $\gamma_{1}$ and $D_{1}$ are defined by:

(2) $$\begin{array}{c} \gamma_{1}=\frac{\bar{T_{N}^{2}}}{\bar{T_{R}^{2}}}, \end{array}$$

(3) $$\begin{array}{c} D_{1}=b\bar{T_{N}^{2}}+\omega \left(\rho_{vN}-a\gamma_{1}\rho_{vR}\right). \end{array}$$

The fraction of vegetation cover (FVC) is represented by *ω*, and the variables $\rho_{R}$ and $\rho_{N}$ represent the TOC reflectance in the red and NIR bands, respectively. The variables $\rho_{vR}$ and $\rho_{vN}$ represent the ’pure’ canopy reflectances independent of the soil surface beneath the canopy layer. Finally, $\bar{T_{R}^{2}}$ and $\bar{T_{N}^{2}}$ represent the area-averaged two-way transmittances ($T_{R}^{2}$ and $T_{N}^{2}$), defined by:

(4) $$\begin{array}{c} \bar{T_{R}^{2}}=\omega T_{R}^{2}+1-\omega , \end{array}$$

(5) $$\begin{array}{c} \bar{T_{N}^{2}}=\omega T_{N}^{2}+1-\omega . \end{array}$$

These variables are explained in additional detail elsewhere [17,21,23].

The second form of the vegetation isoline was derived by only including the interaction terms up to the second-order in the NIR band. The interaction terms in the red band could be expressed using the first-order terms. The asymmetric-order of the approximation in the two bands significantly reduced the error in the isoline relative to the first-order form. The asymmetric form of the vegetation isoline may be written (with the truncation term $\epsilon_{2}$) as

(6) $$\begin{array}{c} \rho_{N}=a^{2}\zeta \rho_{R}^{2}+a\gamma_{2}\rho_{R}+D_{2}+\epsilon_{2}, \end{array}$$

using the definitions:

(7) $$\begin{array}{c} \zeta =\omega T_{N}^{2}R_{vN}/{\left(\bar{T_{R}^{2}}\right)}^{2}, \end{array}$$

(8) $$\begin{array}{c} \gamma_{2}=\gamma_{1}+\delta_{1}, \end{array}$$

(9) $$\begin{array}{c} D_{2}=D_{1}+\delta_{0}, \end{array}$$

(10) $$\begin{array}{c} \delta_{0}=\zeta {\left(b\bar{T_{R}^{2}}-\omega a\rho_{vR}\right)}^{2}, \end{array}$$

(11) $$\begin{array}{c} \delta_{1}=2\zeta \left(b\bar{T_{R}^{2}}-\omega a\rho_{vR}\right). \end{array}$$

The variable $R_{vN}$ represents the bi-hemispherical reflectance of the canopy layers at the bottom surface, which appears only in the NIR band.

### 2.2. Errors in the Vegetation Isoline Equations

**Figure 1:**
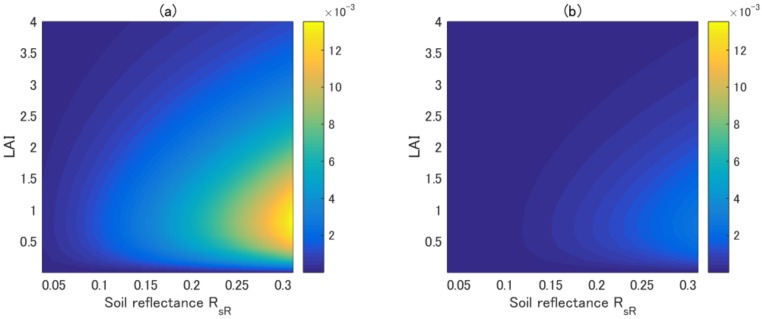
(**a**) Error in the first-order isoline; and (**b**) error in the asymmetric-order isoline. LAI: leaf area index.

The asymmetric approximation of the vegetation isoline achieved greater accuracy than the first-order approximation. This fact could be confirmed by conducting a set of numerical simulations and plotting the errors of the two approximated forms. The errors of the two approximated isolines (the first-order and asymmetric-order approximations) were computed assuming a fully covered vegetation canopy, where the value of FVC was set to unity. The PROSAIL model was used to simulate the TOC reflectance by varying the leaf area index (LAI) and soil reflectance spectra (from dark to bright soil). Figure 1a,b shows plots of the error in the first-order isoline and the asymmetric isoline, respectively, as a function of the LAI and soil reflectance. The error in the first-order isoline reached 0.01 in reflectance units as the soil reflectance increased. By contrast, the error in the asymmetric-order isoline was much smaller than that in the first-order approximation, nearly one order of magnitude smaller, as summarized in our previous study [23].

**Figure 2:**
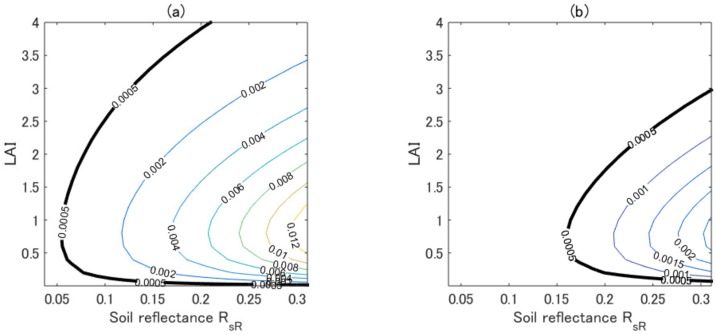
Comparison of the errors in the vegetation isolines with the error (0.0005) computed from the noise corresponding to a signal-to-noise ration (SNR) of 200 at the NIR reflectance of 0.1. (**a**) The error in the first-order isoline; and (**b**) the error in the asymmetric-order isoline. The thick solid lines indicate the contour lines corresponding to 0.0005.

We next focused on testing whether the accuracy of the asymmetric approximation was satisfactory from a parameter retrieval point of view. This point was examined by comparing the isoline errors with an error equivalent to the noise level in the reflectance measurements. The comparison was implemented by assuming a simple scenario such that the value of the SNR in the NIR reflectance was 200 and the average value of the NIR reflectance was 0.1 over the entire parameter range. Although this assumption was made for the sake of simplicity, it was a rather conservative assumption because the averaged NIR reflectance is expected to exceed 0.1 in most cases. Under these assumptions, the noise equivalent error could be obtained as 0.0005 in reflectance units over the entire parameter range. With this quick estimate of the noise equivalent error, Figure 2 shows the contour plots of the errors in the first-order approximation (left) and in the asymmetric-order approximation (right). The contour lines that corresponded to the value of 0.0005 are emphasized by thicker black lines in the figures. These results indicated that the error in the first-order approximated isoline mostly exceeded the noise equivalent error for the majority of the cases (represented by the combinations of the LAI and the soil reflectance). Although the error in the asymmetric-order approximation became much smaller than that of the first-order approximation, the errors still exceeded 0.0005, especially at higher soil reflectances. These results suggested that if the asymmetric-order approximated isoline was used for parameter retrieval, the errors in the retrieved results would be larger than the error introduced by the sensor noise. These results indicated that the accuracy of the asymmetric-order approximation required further improvement.

## 3. Approaches

**Figure 3:**
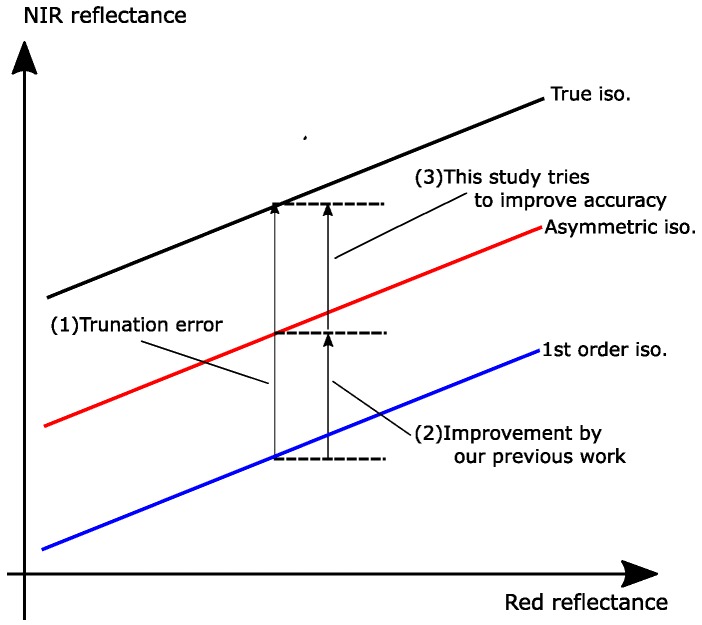
Illustration of the truncation error in the vegetation isoline equations and its improvement by this and previous studies.

Improved accuracy was achieved by including the second-order interaction term only in the NIR band. This modification shifted the first-order approximated isoline upward in the reflectance subspace. Figure 3 illustrates this shifting process and the mechanism by which the accuracy was improved via the asymmetric-order approximation. The degree of shifting from the first-order isoline is illustrated as the difference, along the NIR axis, between the blue line and the red line in the figure. This difference remained smaller than the difference between the first-order isoline and the true vegetation isoline (illustrated as the difference between the blue line and the black line). The gap between the asymmetric-order isoline (red line) and the true isoline (black line) must be minimized to achieve the highest accuracy, which this study attempts to address.

This gap could be analytically evaluated by clarifying the difference between the first-order and the asymmetric-order isoline equations. The asymmetric-order approximation form of the vegetation isoline was obtained by neglecting the higher-order interaction term $\epsilon_{2}$ from Equation (6). The definition of $\gamma_{2}$, Equation (8), was used to express the isoline equation as:

(12) $$\begin{array}{c} \rho_{N}\approx a^{2}\zeta \rho_{R}^{2}+a\gamma_{1}\rho_{R}+a\delta_{1}\rho_{R}+D_{1}+\delta_{0}. \end{array}$$

After rearranging Equation (12) by noting the form of the first-order isoline Equation (1), the above equation could be transformed to

(13) $$\begin{array}{c} \rho_{N}\approx a\gamma_{1}\rho_{R}+D_{1}+\left(a^{2}\zeta \rho_{R}^{2}+a\delta_{1}\rho_{R}+\delta_{0}\right). \end{array}$$

The term in the parentheses on the right-hand-side represents the contribution of the asymmetric second-order term, illustrated as the distance between the blue line and the red line in Figure 3. Equation (13) suggests that an adjustment to this distance (overcorrection term) could fill the gap between the red line and the black line, further improving its accuracy.

One way to adjust the overcorrection term is to introduce a factor into the last term of Equation (13). The factor (represented by *k*) introduced into the last term of Equation (13) could be explicitly introduced in the equation,

(14) $$\begin{array}{c} \rho_{N}\approx a\gamma_{1}\rho_{R}+D_{1}+k\left(a^{2}\zeta \rho_{R}^{2}+a\delta_{1}\rho_{R}+\delta_{0}\right). \end{array}$$

Solving Equation (14) for *k*, we have:

(15) $$\begin{array}{c} k=\frac{\rho_{N}-\left(a\gamma_{1}\rho_{R}+D_{1}\right)}{a^{2}\zeta \rho_{R}^{2}+a\delta_{1}\rho_{R}+\delta_{0}}. \end{array}$$

The value of *k* could be computed from Equation (15) for each combination of the model input parameter used for the reflectance simulation. For example, selecting LAI, FVC, and the soil reflectance of the red band $R_{sR}$ as the set of parameters to be varied during the simulation, with the number of grids for each parameter set to 21, a total of 9261 distinctive values of *k* will be obtained. Because *k* depends on a set of parameters, the most accurate way to adjust this scenario is to model the variations in *k* as a function of all parameters. Such an algorithm, however, is not practical to implement at this stage of investigation because one must estimate all input parameters prior to determining *k*. Specifically, LAI, FVC, and the soil reflectance must be estimated to determine *k*. The adjustment approach may be made more practical by determining the optimum constant for *k* according to the following approach, finding a constant value for *k* that minimizes the error of the adjusted isoline, Equation (14), over the entire range of the input parameters. This constant is considered to be the optimum value of *k*, denoted by $k_{opt}$ in this study.

## 4. Results of the Numerical Simulations

### 4.1. Parameter Settings for the Numerical Experiments

The variables used in a series of numerical simulations were computed using the canopy radiative transfer code, PROSAIL [25], which consists of the leaf optical properties model (PROSPECT) [32] and the canopy reflectance model (SAIL) [33]. The parameter settings in the simulations are summarized in Table 1. LAI, FVC, and the soil brightness (soil factor) were varied in this study. LAI was varied from 0.0 to 4.0 in 0.2 increments (21 intervals). The soil factor was varied from 0.0 to 1.0 in 0.05 increments (21 variations), which were used to change the mixture ratio of the reflectance spectra of the wet and dry soil provided with the code. The canopy reflectance spectra obtained using PROSAIL were linearly mixed with the soil spectra using the fraction of vegetation cover (FVC), *ω*, as the weight that was varied from 0.0 to 1.0 in 0.05 increments (21 intervals). The results section focuses on the use of a Spherical model to represent the leaf angle distribution (LAD), with the exception of the simulations presented in Section 4.6, which employs five LAD models (planophile, erectophile, plagiophile, extremophile, and uniform) to examine the effects of the LAD on our simulations. The input parameters in PROSAIL, including the other parameters fixed in this study, are listed in Table 1. The total number of spectra was 9261 (21 × 21 × 21) for a single LAD. (The parameter grids are finer than in our previous study [23].) We employed 655 nm and 865 nm reflectance spectra for the red and NIR wavelength regions, which corresponded to the center of the red and NIR bands in the Landsat 8 Operational Land Imager (OLI).

### 4.2. Numerical Procedure Used for the Isoline Parameter Retrieval

The parameters in the isoline equations were computed according to the procedures reported previously [23,26]. $T_{v\lambda }^{2}$ and $\rho_{v\lambda }$ were determined based on two hypothetical simulations. First, $\rho_{v\lambda }$ was computed using spectrally flat zero reflectances of the soil surface. Subsequently, $T_{v\lambda }$ was approximated using simulated reflectances and a median reflectance of the soil surface, and $\rho_{v\lambda }$ was computed in previous step [26]. The parameter $R_{vN}$, which was required for the computation of *ξ*, was obtained by conducting an additional simulation in which the soil spectrum was even brighter than was assumed in the simulation used to compute $T_{v\lambda }$. The soil spectrum was also spectrally flat in this case [23]. In the simulation, the TOC canopy reflectances in the NIR were approximated using first- and second-order interaction terms between the canopy layer and the soil surface,

(16) $$\begin{array}{c} \rho_{N}\approx \omega \rho_{vN}+\bar{T_{N}^{2}}R_{sN}+\omega T_{N}^{2}R_{vN}R_{sN}^{2}. \end{array}$$

where $R_{sN}$ represents the bi-hemispherical reflectance of the soil surface for the NIR band. $R_{vN}$ was then derived by solving Equation (16) for $R_{vN}$. The isoline parameters for the canopy layer were obtained using these variables. The slope and offset of the soil line equation over the red and NIR reflectance spaces used in the isoline parameters were obtained from a linear regression of the reflectance spectra for the wet and dry soils, shown in Table 1 (a=1.24 and b=0.026).

### 4.3. Variations in k

**Figure 4:**
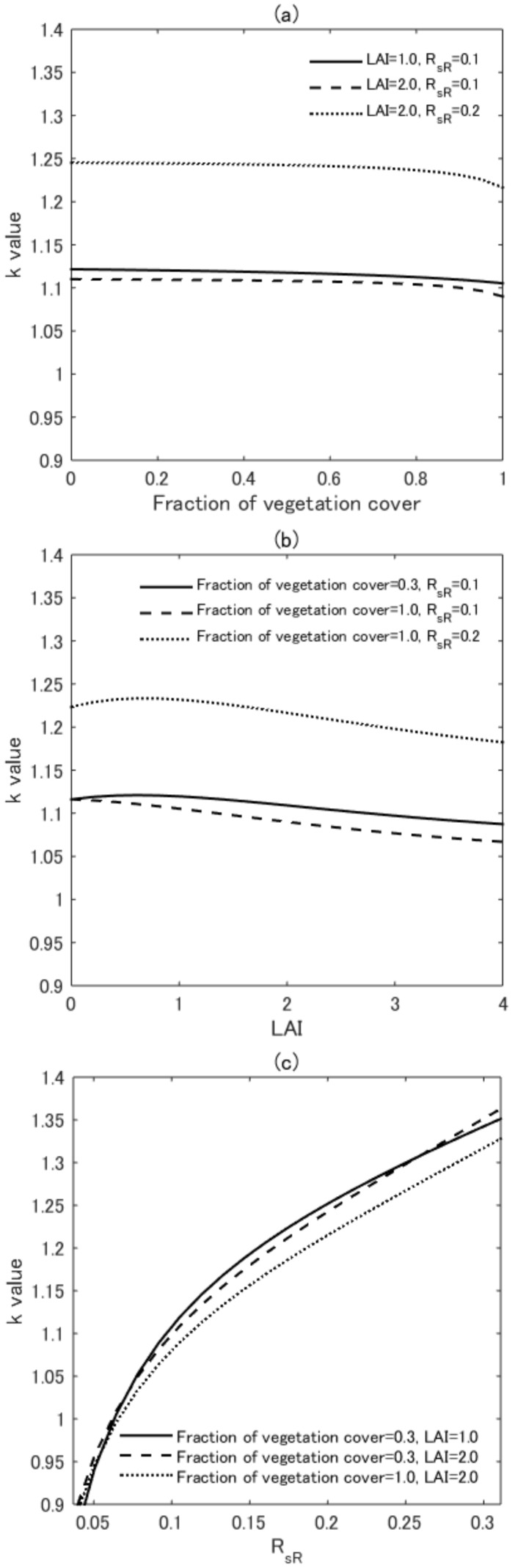
(**a**) Plot of the *k*-values along with the FVC over three pairs of fixed LAI and $R_{sR}$ (LAI = 1.0 and $R_{sR}$ = 0.1, LAI = 2.0 and $R_{sR}$ = 0.1, and LAI = 2.0 and $R_{sR}$ = 0.2); (**b**) Plot of the *k*-values along with LAI over three pairs of fixed FVC and $R_{sR}$ (FVC = 0.3 and $R_{sR}$ = 0.1, FVC = 1.0 and $R_{sR}$ = 0.1, and FVC = 1.0 and $R_{sR}$ = 0.2); (**c**) Plot of the *k*-values along with $R_{sR}$ over three pairs of fixed FVC and LAI (FVC = 0.3 and LAI = 1.0, FVC = 0.3 and LAI = 2.0, and FVC = 1.0 and LAI = 2.0).

The dependences of LAI, FVC, and $R_{sR}$ on *k* were analyzed based on numerical experiments in which the *k*-value was computed in the previous step using Equation (15). Three experimental conditions were applied to compute *k*: (1) FVC was varied using three pairs of fixed LAI and $R_{sR}$ values; (2) LAI was varied using three pairs of fixed FVC and $R_{sR}$ values; and (3) $R_{sR}$ was varied using three pairs of fixed FVC and LAI values. The results of the first case are shown in Figure 4a. The *k*-values are plotted against FVC for “LAI = 1.0 and $R_{sR}$ = 0.1”, “LAI = 2.0 and $R_{sR}$ = 0.1”, and “LAI = 2.0 and $R_{sR}$ = 0.2”, denoted by the solid, dashed, and dotted lines, respectively. The *k*-values were relatively insensitive to the changes in FVC. The differences between the *k* values for FVC = 0.0 and FVC = 0.9 were less than 1%, and the differences for FVC = 0.0 and FVC = 1.0 were less than 3% for each pair of LAI and $R_{sR}$. The strong dependence of $R_{sR}$ on *k* was identified from the large differences between the *k*-value curves obtained at $R_{sR}$ = 0.1 and = 0.2.

Similarly, the *k*-values were relatively insensitive to LAI, as shown in Figure 4b (results are shown for the second experimental case). These *k*-values are plotted against LAI for “FVC = 0.3 and $R_{sR}$ = 0.1”, “FVC = 1.0 and $R_{sR}$ = 0.1”, and “FVC = 1.0 and $R_{sR}$ = 0.2”, respectively. The differences between the *k*-values for LAI = 0.0 and LAI = 4.0 were less than 5%. The differences between the results obtained for $R_{sR}$ = 0.1 and = 0.2 were similarly large, as shown in Figure 4a.

Figure 4c presents the results obtained from the third case, which described the *k*-values along $R_{sR}$ for “FVC = 0.3 and LAI = 1.0”, “FVC = 0.3 and LAI = 2.0”, and “FVC = 1.0 and LAI = 2.0”, respectively. The *k*-values showed an approximately 50% increase (from 0.9 to 1.35) with increasing $R_{sR}$. The differences among the three pairs of LAI and FVC affected the *k*-value to a much smaller degree than did the differences between the maximum and minimum $R_{sR}$ values. These results indicated that the *k*-values depended heavily on $R_{sR}$ but were nearly independent of FVC and LAI (greenness level of the vegetation canopy).

### 4.4. Optimum k-Values ($k_{opt}$)

**Figure 5:**
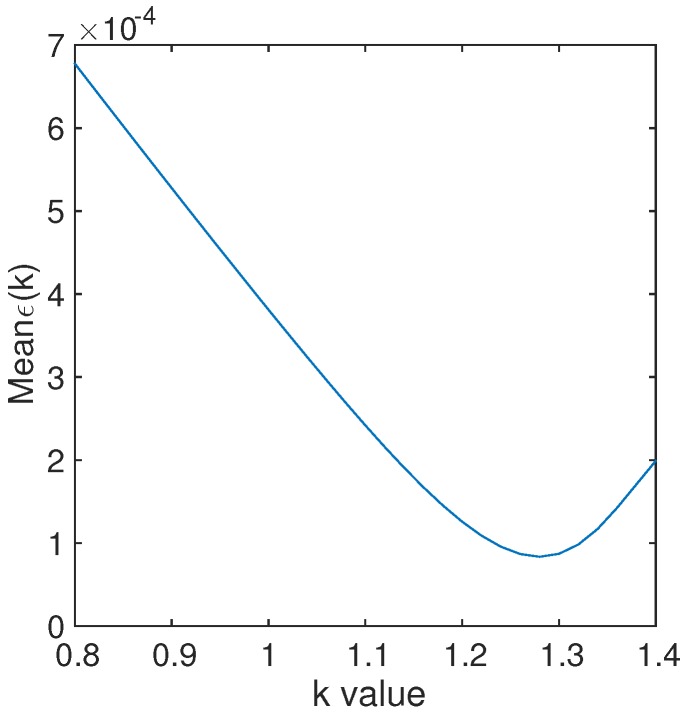
Plot of the mean value of *ϵ* versus the *k*-value.

The *k*-values were computed using all possible pairs of the input parameters (FVC, LAI, and $R_{sR}$ were changed; LAD was fixed to a spherical model; and all other input parameters were fixed, as shown in Table 1). The optimum value of *k* was then determined by computing the distances (*ϵ*) as the errors between the true spectra ***ρ*** (including all the higher-order terms) and the vegetation isolines (the adjusted asymmetric isolines by Equation (14)),

(17) $$\begin{array}{c} \epsilon (k)=min(∥\rho -\hat{\rho }\left(k\right){∥}_{2}), \end{array}$$

where $\hat{\rho }\left(k\right)$ denotes the spectra on the vegetation isolines for the *k*-value as the input. Note that ϵ(k) for k=0 and k=1 corresponds to the error of the first-order vegetation isoline and the asymmetric-order vegetation isoline without optimization, respectively. More than 9261 values of *k* were obtained using Equation (15), and each *k* was used to compute *ϵ* for 9261 patterns of the reflectance spectra. A two-dimensional array of *ϵ* values with a size of 9261 (spectral dimension) × 9261 (*k*-value dimension) was obtained. Figure 5 plots the values of *ϵ* averaged along the spectral dimensions as a function of the *k*-values. The error *ϵ* decreased until the *k*-value reached 1.25–1.30 and changed to an increasing function upon further increases in the *k*-value.

The optimum values of *k*, $k_{opt}$ were identified as follows: Six variations in the *k*-value were assumed: 1.25, 1.26, 1.27, 1.28, 1.29, and 1.30; the errors were approximated using the 9261 spectral patterns, that is, *ϵ* were computed for each *k*-value. The mean, standard deviation (STD), and maximum of *ϵ* for each *k*-value were computed and are summarized in Table 2. The minimum values of the mean *ϵ* were 8.35 ×$10^{-5}$ for *k* = 1.28. The values of the STD for *k* = 1.29 were, however, smaller than those obtained for *k* = 1.28. The same applied to the maximum. Accordingly, the optimum *k*-value, $k_{opt}$, was determined to be 1.29 for the Spherical LAD in this study.

### 4.5. Evaluation of $k_{opt}$ = 1.29

**Figure 6:**
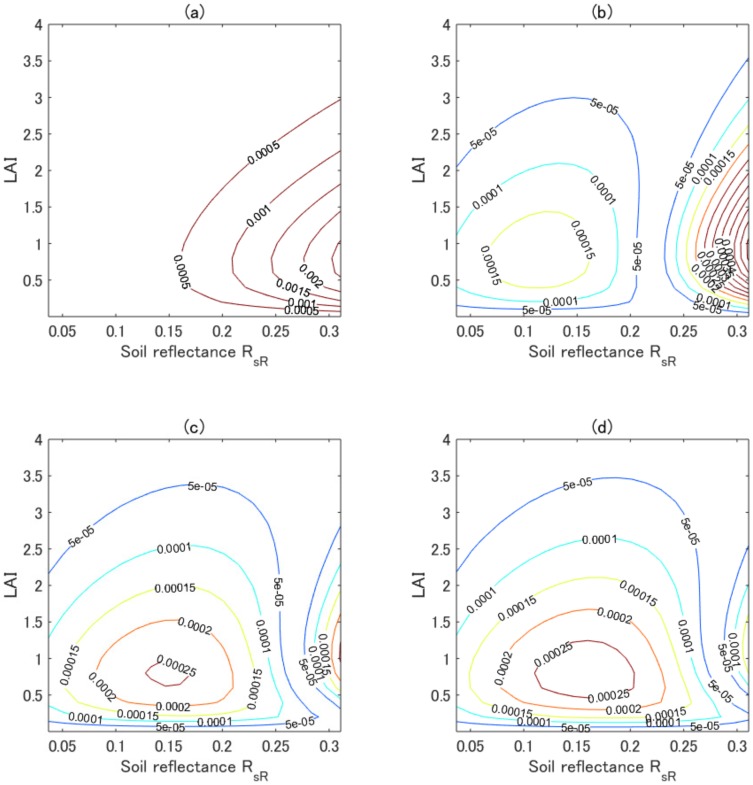
(**a**) Contour plot of *ϵ* over LAI and $R_{sR}$ space for *k* = 1.00; (**b**) Contour plot of *ϵ* for *k* = 1.25; (**c**) Contour plot of *ϵ* for *k* = 1.29; (**d**) Contour plot of *ϵ* for *k* = 1.30.

The validity of using $k_{opt}$ = 1.29 was then evaluated using contour plots of *ϵ* over LAI and $R_{sR}$ space, holding FVC fixed at unity. Four variations of *k* (1.0, 1.25, 1.29, and 1.30) were considered. In Figure 6a, the minimum value of *ϵ* for k=1.0 was 0.0002, and the errors were greater than those obtained under other conditions, as shown in Figure 6. Figure 6b presents results obtained for *k* = 1.25 and reveals that *ϵ* was less than 0.00015 for $R_{sR}<$0.26. *ϵ*, however, it increased with increasing $R_{sR}$, especially for $R_{sR}>$ 0.26 and for LAI approaching 1.0. Figure 6c shows that for *k* = 1.29, the maximum of *ϵ* was approximately 0.00025. Overall, *ϵ* was small across the entire parameter space. The results of *ϵ* obtained for *k* = 1.30 were slightly larger than the values obtained for *k* = 1.29 although *ϵ* was small for $R_{sR}>$ 0.26.

The error across the entire parameter space (e.g., the mean value of *ϵ*) was smallest for k=1.29 (Figure 6c and Table 2), although *ϵ* for *k* = 1.25 was less than the value obtained for other *k*-values for $R_{sR}<$ 0.26 (Figure 6b), and *ϵ* was smallest for *k* = 1.30 for $R_{sR}>$ 0.26 (Figure 6d). This experiment, therefore, validated the use of the optimum *k*-value, $k_{opt}$ (=1.29) for minimizing the overall error in the predicted NIR reflectances based on the adjusted asymmetric isoline equation. Furthermore, the value of *ϵ* for $k_{opt}$ = 1.29, as shown in Figure 6c, was small relative to the noise equivalent error (0.0005).

We next computed the statistical profile of the errors in the first-order, the asymmetric-order, and the adjusted asymmetric-order ($k_{opt}$ = 1.29) isoline equations. Table 3 lists the mean, STD, and maximum approximation error in the isoline equations. The mean values of the errors for the adjusted asymmetric-order isoline equations were reduced to 4% and 22% of the value obtained from the first-order and the asymmetric-order isoline equations. Likewise, the STD and maximum of the errors in the adjusted asymmetric-order isoline equations were much smaller than those obtained from other isoline equations. The statistical distribution of the errors in the adjusted asymmetric isoline equations did not exceed the noise equivalent errors (0.0005), even in the case of the maximum error.

### 4.6. Evaluation of $k_{opt}$ = 1.29 for Various LADs and Variations in the Optimum k-Value

The performances of the derived isoline equations for $k_{opt}$ = 1.29 were evaluated over various LADs in PROSAIL, including planophile, erectophile, plagiophile, extremophile, and uniform distributions, respectively. Table 4 lists the statistical analysis associated with approximating errors in the isoline equations (mean, STD, and maximum). The statistical distribution of the planophile was nearly identical to that of the Spherical model, as shown in Table 3. In other LADs, the statistical distributions of the derived equations were nearly equal to or more than half of the corresponding distributions of the other isoline equations. Also, although the maximum errors could exceed the noise equivalent error (0.0005), the mean values of the errors were less than 0.0004 for all LADs. This fact indicated that the adjusted asymmetric isoline equations with $k_{opt}$ = 1.29 provided acceptable results, regardless of the choice of LAD.

**Figure 7:**
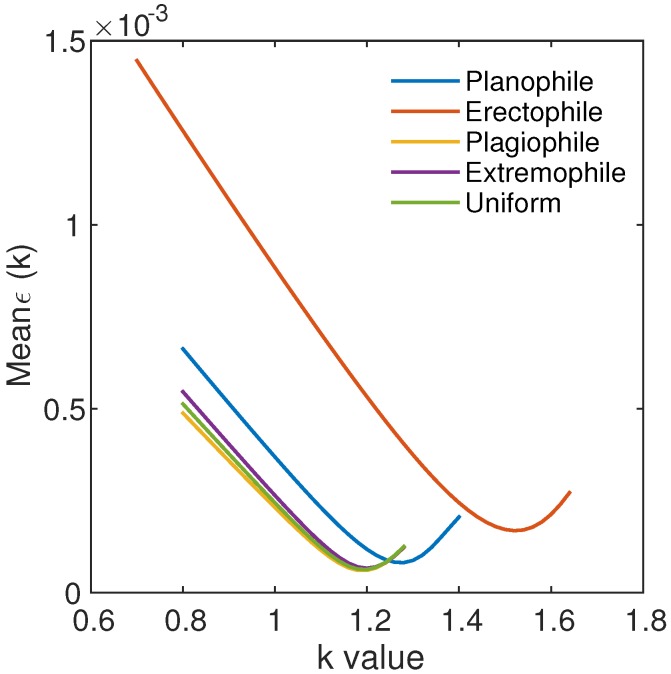
Plot of the mean *ϵ* as a function of the *k*-value for the planophile, erectophile, plagiophile, extremophile, and uniform LADs, respectively.

The optimum values of *k* for the various LADs were explored using the algorithm presented in Section 4.4. The approximation errors *ϵ* with size of 9261 (spectral dimension) × 9261 (*k*-value dimension) were computed for each LAD, and the values of *ϵ* averaged along the spectral dimensions were computed and plotted as a function of the *k*-value for each LAD. Figure 7 plots the mean *ϵ* versus *k*-value for the various LADs. The *k*-values that provided the minimum value of the mean *ϵ* were approximately 1.2–1.3, except for the erectophile model, indicating that the minimum value of the mean *ϵ* occurred for k>1.5.

Table 5 lists the optimum *k*-value and mean, STD, and maximum *ϵ*, where ${\hat{\rho }}_{N}$ was computed using the optimum *k*-value for each LAD obtained in our simulations. The mean values of the errors were approximately equal to or smaller than 0.0001. The magnitude of the STD of the errors was similar to that of mean. The maximum value of the errors was less than the noise equivalent error (0.0005), except for the erectophile model. The appropriate selection of the optimum *k*-value thus led to an accurate prediction of the NIR reflectances, but the use of $k_{opt}$ = 1.29 provided an acceptable accuracy in terms of the SNR, even though this accuracy was not optimal for each LAD.

### 4.7. Comparison with the Noise-Equivalent Errors in Satellite Sensors

This study sought to decrease the errors associated with predicting the NIR reflectances by using red reflectances in the vegetation isoline equations. The goal was to decrease the errors to the level of the intrinsic errors of the sensor SNR values. Therefore, the errors in the first-order, the asymmetric-order, and the adjusted asymmetric isoline equations with $k_{opt}$ = 1.29 were compared with the error arising from the SNR of the earth observation sensors currently in space orbit. We employed the SNRs of four sensors, including the Aqua-Moderate Resolution Imaging Spectroradiometer (MODIS) [34], the Landsat 8-Operational Land Imager (OLI) [35], the Greenhouse Gases Observing Satellite (GOSAT)-Cloud and Aerosol Imager (CAI) [36], and the Suomi National Polar-orbiting Partnership (SNPP)-Visible Infrared Imaging Radiometer Suite (VIIRS) [37], as summarized in Table 6.

The ratio of the relative errors in the isoline equations to the sensor SNR (*r*) was computed according to:

(18) $$\begin{array}{c} r=\frac{\epsilon (k)}{\left(\rho_{N}/SNR\right)} \end{array}$$

The isoline equations were superior to the noise equivalent errors for values of less than unity, whereas the equations were inferior to the noise equivalent errors for values greater than unity. For comparison, the FVC and LAD were fixed, respectively, to unity and spherical.

**Figure 8:**
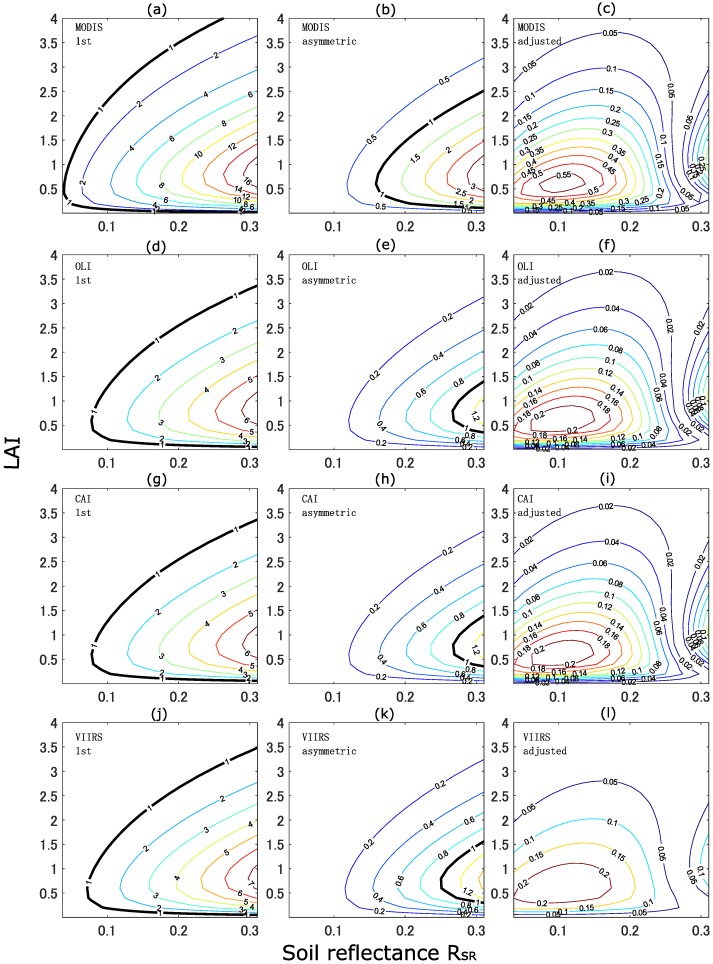
Contour plots of *r* over LAI-$R_{sr}$ space. From the top to the bottom, each plot correspond to the MODIS (a, b, c), OLI (d, e, f), CAI (g, h, i), and VIIRS (j, k, l) sensors, respectively. From the left to right column, each plot corresponds to the first-order (a, d, g, j), asymmetric-order (b, e, h, k), and adjusted asymmetric-order (c, f, i, l) (with $k_{opt}$ = 1.29) isoline equations, respectively. The bold line indicates *r* = 1.0. (**a**) MODIS-first; (**b**) MODIS-asymmetric; (**c**) MODIS-adjusted; (**d**) OLI-first; (**e**) OLI-asymmetric; (**f**) OLI-adjusted; (**g**) CAI-first; (**h**) CAI-asymmetric; (**i**) CAI-adjusted; (**j**) VIIRS-first; (**k**) VIIRS-asymmetric; (**l**) VIIRS-adjusted.

Figure 8 plots *r* over the LAI-$R_{sR}$ space. Thicker black lines correspond to *r* = 1.0. MODIS, OLI, CAI, and VIIRS correspond to the four rows of Figure 8 from the top to the bottom. From the left to the right column, the results of the first-order, asymmetric-order, and adjusted asymmetric-order isoline equations are plotted. The results of first-order isoline equations (Figure 8a,d,g,j) indicate that *r* exceeded unity over a large area of parameter space. The asymmetric-order isoline equations resulted in smaller values of *r* and exceeded unity for relatively large values of $R_{sR}$; however, the areas of these parts were significantly smaller than the area observed in the first-order isoline, as shown in Figure 8b,e,h,k. Finally, the results of *r* obtained from the adjusted asymmetric-order isoline equations (Figure 8c,f,i,l) revealed that *r* never exceeded unity, and the maximum values of *r* were less than 0.5 for all sensors. In summary, the errors in the adjusted asymmetric isoline equations with $k_{opt}$ = 1.29 were smaller than the error arising from the SNR of the four earth observation sensors.

## 5. Discussion and Conclusions

The asymmetric-order isoline equations, derived from a previous study, were reformulated as first-order isoline equations plus a correction term multiplied by a parameter *k*. The derived equations with optimized *k* (as a constant) improved the accuracy of the asymmetric-order isoline equations while retaining the simplicity of the equations. The *k*-value was assumed to be a function of LAI, FVC, and $R_{sR}$, and the influences of $R_{sR}$ on *k* were much greater than the influences of LAI and FVC such that *k* could be considered primarily to be a function of $R_{sR}$. One advantage of the isoline equations was that the parameters in the equations were independent of the soil brightness, i.e., $R_{sR}$; therefore, we fixed the *k*-value to an optimum instead of varying this parameter as a function of $R_{sR}$.

The errors in the adjusted asymmetric-order isoline equations were computed using $k_{opt}$. The errors in the reflectances predicted by the adjusted asymmetric-order isoline equations with $k_{opt}$ were 4% and 22% of the errors predicted using the first-order and asymmetric-order isoline equations, respectively.

The value of $k_{opt}$ was optimal for the Spherical LAD. The adjusted asymmetric-order isoline equations with $k_{opt}$ reduced the errors significantly in the reflectances calculated using any of the six LADs defined in this study (less than half of the errors for the asymmetric-order isoline equations), although the use of the optimal *k*-value along with each LAD reduced the errors more significantly. In addition, the errors in the adjusted asymmetric-order isoline equations were small over the entire parameter space relative to the noise equivalent errors computed from the SNR of the satellite sensors currently in orbit (Aqua-MODIS, Landsat 8-OLI, SNPP-VIIRS, and GOSAT-CAI). The first-order and asymmetric-order isoline equations displayed both superiority and inferiority to the noise equivalent errors by relying on the canopy and soil conditions.

This study achieved its goal of reducing the error in the adjusted asymmetric-order isoline equations using a fixed *k*-value, yielding an error that was less than the noise equivalent errors based on the SNRs of some major satellite sensors, without complicating the isoline equations. Validation of the derived equations would require additional numerical experiments involving the application of other radiative transfer models of the vegetation canopy. Improved accuracy in the equations may be necessary if the sensor’s SNR were to increase as a result of technological advancements in the sensor instrument design.

## Figures and Tables

**Table 1:** Input parameters used in the numerical simulations.

Geometry	
Solar zenith angle	$30^{\circ }$
Observation zenith angle	$10^{\circ }$
Relative azimuth angle	$0^{\circ }$
**Pixel Heterogeneous Property**	
Fraction of vegetation cover (FVC)	0.0–1.0
**Canopy Properties**	
Leaf area index (LAI)	0.0–4.0
Hotspot size parameter	0.01
**Leaf Structural and Chemical Properties**	
Leaf angle distribution (LAD)	Spherical, Planophile, Erectophile
	Plagiophile, Extremophile, Uniform
Leaf mesophyll structure	1.5
Chlorophyll-a and -b	40 $\mu g/cm^{2}$
Carotenoid content	8 $\mu g/cm^{2}$
Leaf mass per area	0.009 $g/cm^{2}$
Equivalent water thickness	0.01 cm
Brown pigment content	0
**Soil Properties**	
Wet soil reflectances at 655 and 865 nm	0.037 and 0.071
Dry soil reflectances at 655 and 865 nm	0.311 and 0.412
Soil factor (mixture ratio of wet and dry soils)	0.0–1.0 [0.0: wet soil; 1.0: dry soil]

**Table 2:** Statistics of ϵ(k) for k = 1.25, 1.26, 1.27, 1.28, 1.29, and 1.30. STD: standard deviation.

	LAD: Spherical					
	1.25	1.26	1.27	1.28	1.29	1.30
Mean	$9.06\times 10^{-5}$	$8.68\times 10^{-5}$	$8.44\times 10^{-5}$	$8.35\times 10^{-5}$	$8.43\times 10^{-5}$	$8.71\times 10^{-5}$
STD	$1.08\times 10^{-4}$	$9.54\times 10^{-5}$	$8.44\times 10^{-5}$	$7.58\times 10^{-5}$	$7.05\times 10^{-5}$	$6.89\times 10^{-5}$
MAX	$7.05\times 10^{-4}$	$6.36\times 10^{-4}$	$5.66\times 10^{-4}$	$4.97\times 10^{-4}$	$4.31\times 10^{-4}$	$3.66\times 10^{-4}$

**Table 3:** Statistics of the errors in first-order, asymmetric-order, and adjusted asymmetric-order ($k_{opt}$ = 1.29) isoline equations.

	LAD: Spherical				
	**First-Order**	**Asymmetric**	**Adjusted Asymmetric**	**adj./first**	**adj./asym.**
Mean	$2.10\times 10^{-3}$	$3.81\times 10^{-4}$	$8.43\times 10^{-5}$	4.0%	22.1%
STD	$2.43\times 10^{-3}$	$5.06\times 10^{-4}$	$7.05\times 10^{-5}$	2.9%	13.9%
MAX	$1.35\times 10^{-2}$	$2.67\times 10^{-3}$	$4.31\times 10^{-4}$	3.2%	16.1%

**Table 4:** Statistical distributions of the errors in the first-order, asymmetric-order, and adjusted aymmetric-order ($k_{opt}$ = 1.29) isoline equations for the five LADs, including planophile, erectophile, plagiophile, extremophile, and uniform distributions.

	LAD: Planophile				
	first-order isoline	asymmetric isoline	adjusted isoline	adj./1st	adj./asym.
Mean	$2.07\times 10^{-3}$	$3.69\times 10^{-4}$	$8.39\times 10^{-5}$	4.1%	22.7%
STD	$2.40\times 10^{-3}$	$4.91\times 10^{-4}$	$6.76\times 10^{-5}$	2.8%	13.8%
MAX	$1.34\times 10^{-2}$	$2.59\times 10^{-3}$	$3.79\times 10^{-4}$	2.8%	14.6%
	**LAD: Erectophile**				
	first-order isoline	asymmetric isoline	adjusted isoline	adj./1st	adj./asym.
Mean	$3.08\times 10^{-3}$	$8.83\times 10^{-4}$	$3.89\times 10^{-4}$	12.6%	44.1%
STD	$3.54\times 10^{-3}$	$1.12\times 10^{-3}$	$5.53\times 10^{-4}$	15.6%	49.4%
MAX	$1.87\times 10^{-2}$	$5.79\times 10^{-3}$	$2.95\times 10^{-3}$	15.8%	50.9%
	**LAD: Plagiophile**				
	first-order isoline	asymmetric isoline	adjusted isoline	adj./1st	adj./asym.
Mean	$1.71\times 10^{-3}$	$2.31\times 10^{-4}$	$1.35\times 10^{-4}$	7.9%	58.4%
STD	$2.00\times 10^{-3}$	$3.15\times 10^{-4}$	$1.24\times 10^{-4}$	6.2%	39.4%
MAX	$1.13\times 10^{-2}$	$1.62\times 10^{-3}$	$7.78\times 10^{-4}$	6.9%	48.0%
	**LAD: Extremophile**				
	first-order isoline	asymmetric isoline	adjusted isoline	adj./1st	adj./asym.
Mean	$1.89\times 10^{-3}$	$2.64\times 10^{-4}$	$1.37\times 10^{-4}$	7.2%	51.9%
STD	$2.19\times 10^{-3}$	$3.57\times 10^{-4}$	$1.20\times 10^{-4}$	5.5%	33.6%
MAX	$1.23\times 10^{-2}$	$1.84\times 10^{-3}$	$7.04\times 10^{-4}$	5.7%	38.3%
	**LAD: Uniform**				
	first-order isoline	asymmetric isoline	adjusted isoline	adj./1st	adj./asym.
Mean	$1.79\times 10^{-3}$	$2.44\times 10^{-4}$	$1.38\times 10^{-4}$	7.7%	56.6%
STD	$2.09\times 10^{-3}$	$3.32\times 10^{-4}$	$1.24\times 10^{-4}$	5.9%	37.3%
MAX	$1.17\times 10^{-2}$	$1.71\times 10^{-3}$	$7.60\times 10^{-4}$	6.5%	44.4%

**Table 5:** Optimum *k*-value and statistical distributions of the errors in the adjusted asymmetric isoline equations, with the optimal *k*-values for each of the five LADs, including the planophile, erectophile, plagiophile, extremophile, and uniform distributions.

LAD	Optimum *k*	Mean	STD	MAX
Planophile	1.28	$8.17\times 10^{-5}$	$7.03\times 10^{-5}$	$4.44\times 10^{-4}$
Erectophile	1.53	$1.69\times 10^{-4}$	$1.39\times 10^{-4}$	$8.31\times 10^{-4}$
Plagiophile	1.19	$5.99\times 10^{-5}$	$6.23\times 10^{-5}$	$4.08\times 10^{-4}$
Extremophile	1.2	$6.65\times 10^{-5}$	$6.67\times 10^{-5}$	$4.40\times 10^{-4}$
Uniform	1.20	$6.31\times 10^{-5}$	$6.01\times 10^{-5}$	$3.81\times 10^{-4}$

**Table 6:** SNR in the red and NIR bands for the Aqua-Moderate Resolution Imaging Spectroradiometer (Aqua-MODIS) [38], Landsat 8-Operational Land Imager (Landsat 8 OLI) [39], Greenhouse Gases Observing Satellite (GOSAT)-Cloud and Aerosol Imager (CAI) [40], and Suomi National Polar-orbiting Partnership (NPP)-Visible Infrared Imaging Radiometer Suite (VIIRS) [41]. SNR for MODIS band1 (red) was derived by calculating 128 (sensor design requirement) × 1.57 (ratio of measured SNR in-orbit to sensor design requirement) and SNR for MODIS band 2 (NIR) was derived by 201 × 2.64 [38]. Similaly, SNR for VIIRS I1 and I2 bands (red and NIR) were derived by caluculating 119 × 1.76 and 150 × 1.5, respectively [41].

	MODIS	Landsat8 OLI	GOSAT-CAI	VIIRS
Red band	201	227	200	209
NIR band	530	201	200	225
